# Demographics, Clinical Characteristics, and a Stage‐Based Analysis of Treatments and Outcomes for Squamous Cell Carcinoma of the Penis

**DOI:** 10.1002/cnr2.70383

**Published:** 2025-12-03

**Authors:** George M. Edwards, Lucas W. Ashley, Austin Holmes, Andrew W. Ju, Arjun Bhatt, Michael C. Larkins

**Affiliations:** ^1^ Brody School of Medicine East Carolina University Greenville North Carolina USA; ^2^ Independent Researcher Raleigh North Carolina USA; ^3^ Department of Radiation Oncology, Brody School of Medicine East Carolina University Greenville North Carolina USA; ^4^ Department of Emergency Medicine Boonshoft School of Medicine at Wright State University Fairborn Ohio USA

**Keywords:** lymph node biopsy, lymph node dissection, penile cancer, SEER database, squamous cell carcinoma

## Abstract

**Objectives:**

Penile squamous cell carcinoma (PSCC) is the most common penile cancer, accounting for ≥ 95% of cases, though it accounts for < 1% of all malignancies in men in the United States. We report an updated, stage‐stratified analysis of the efficacy of surgery, radiation, and chemotherapy, including adjuvant and neoadjuvant chemoradiation, with further analysis of demographic and clinical factors.

**Methods:**

Using the Surveillance, Epidemiology, and End Results (SEER) database, patients with PSCC diagnosed between 2000 and 2018 were identified. Five‐year overall survival Cox regression analysis as well as univariate Kaplan–Meier analysis were performed, stratified by demographic and treatment variables.

**Results:**

Two thousand seven hundred eight patients with PSCC were identified, with 57.8% being older than 65 years at diagnosis and 94.2% undergoing surgical intervention. With multivariate analysis, increasing disease stage (*p* < 0.001), age < 65 years (*p* < 0.001), lower disease grade (*p* < 0.001) were all associated with increased survival, while treatment with chemotherapy or radiotherapy was both associated with decreased survival (*p* = 0.002 and < 0.001, respectively). On univariate analysis, less invasive surgery was associated with increased survival among patients with low‐grade, local (*p* < 0.001) or regional (*p* = 0.03) disease. Among those with high‐grade disease, local excision was associated with increased survival (*p* = 0.008), though among those with regional disease no survival difference was seen (*p* = 0.86). Patients with regional disease saw increasing survival with four or more lymph nodes dissected (69% vs. 61%, respectively; *p* = 0.002).

**Conclusions:**

Surgical management of penile SCC remains the mainstay treatment, and less invasive surgery is associated with noninferior or improved 5‐year overall survival regardless of disease stage and grade. Patients with regional disease had increased survival when four or more lymph nodes were dissected. Future analysis of these trends stratified by disease subhistology and more granular analysis of the role of lymphadenectomy are warranted.

## 1 Introduction

Penile cancer is primarily a disease of the developing world, representing up to 10% of male cancers [1]; within the United States though, this disease accounts for < 1% of all malignancies in men [2]. Furthermore, ≥ 95% of all penile cancers are PSCC [2]. Unlike other cancers, penile cancer has not shown improvements in survival on a population level in the United States and Europe since 1990 [3]. Without treatment, patients diagnosed with PSCC generally die within 2 years of diagnosis of the primary lesion due to uncontrolled regional disease or distant metastases [4]. Considered a relatively rare disease, only 26 000 cases have been reported worldwide as of 2022, though it should be noted that given the sensitive nature of this disease, significant underreporting may be present [5]. It should be noted that the exact tumorigenesis of penile SCC is not fully known, though factors including hygiene practices, genetics, exposure to human papillomavirus (HPV), lifestyle, and social determinants of health have all been implicated.

Lymphatic spread is the strongest prognostic indicator for overall survival of PSCC patients [6]. Patients with a single inguinal node involved have a 5‐year survival of approximately 80%, whereas those with involvement of deeper pelvic nodes have a 5‐year survival between 0% and 12% [1]. Preoperative Staging for penile cancer is performed with lymphoscintigraphy or dynamic sentinel lymph node biopsy (DSNB), the former with a reported nodal detection sensitivity of 86% [7] and a range of 78%–88% [8] respectively; specificities have been reported at 26% for lymphoscintigraphy and 100% for DSNB. More recent literature quotes sensitivities around 77% and specificities around 100%, though this may be institution‐dependent [9]. Presentation rates for clinical Staging of lymph nodes have been reported at 43.4%, 24.7%, 15.4%, and 15.4%, for cN0, cN1, cN2, and cN3, respectively [10]. A quarter of patients with Stage cN0 have micrometastatic disease to regional lymph nodes [1].

Surgical resection depends on tumor stage, grade, location, and functional status of the patient. Treatment options for patients with noninvasive or in situ disease include topical therapy, wide local excision, laser therapy, and glansectomy [11]. Mohs micrographic surgery has gained popularity for tumors of earlier stage and grade due to maximized tissue preservation and the ability to visualize the tumor margins [1]. However, tumors of more advanced stage and grade may warrant partial or total penectomy [1]. Pelvic lymphadenectomy is performed at Stage cN3 [12]. The presence and extent of metastasis to the inguinal region have been reported as important predictors of mortality among patients with penile SCC; inguinal lymphadenectomy can be curative and in appropriate patients is the recommended treatment [13]. It should be noted that penile SCC especially compared to other cancers has been reported to have a prolonged loco‐regional phase before distant metastasis occurs, making management of affected lymph nodes appropriate and attractive. Patients with pelvic lymph node involvement have worse 5‐year survival outcomes than patients with solely inguinal lymph node involvement, at 33% vs. 71%, respectively [12]. Traditionally open inguinal lymphadenectomy has been performed, though more recently evidence has emerged that video endoscopic surgery is non‐inferior [13].

For T1‐2 N0 tumors < 4 cm, radiation and chemoradiation can be used as definitive therapy [14]. Such tumors ≥ 4 cm can receive primary radiation or chemoradiation following circumcision [15, 16, 17]. For patients with unresectable disease, namely T3–T4 or positive lymph nodes, circumcision followed by external beam radiation therapy (EBRT) is acceptable, as is postsurgical EBRT in the case of positive margins [11]. While radiation can preserve the structural integrity of the surrounding region, functional impairments of the corpus cavernosa are common, and 10%–35% of patients develop urinary impairments secondary to meatal stenosis [18]. Additionally, radiation may not be effective for tumors with extranodal extension [19]. Neoadjuvant chemotherapy with paclitaxel, ifosfamide, or cisplatin, also known as TIP, has been demonstrated in some populations to prolong survival [20]. Insufficient data are available on the use of adjuvant chemotherapy, though TIP or 5‐FU and cisplatin are accepted regimens [11]. Both radiation and chemotherapy can also be used as palliative options.

Numerous social factors have also been shown to serve as prognostic factors for penile SCC [21]. Several reports have demonstrated that being married is a protective social factor for the development and prognosis of penile SCC [22, 23]. Studies postulate this may be related to exposure to Human Papilloma Virus (HPV) as well as altered hygiene practices among those unmarried versus married who develop SCC of the penis.

Two stage‐stratified PSCC studies were recently published in the Fall of 2023 using the National Cancer Institute's Surveillance, Epidemiology, and End Results (SEER) database [24]—one reported differences in 5‐year survival based on tumor stage [25] and the other reported ranges of local tumor destruction, partial penectomy, radical penectomy, and cancer‐specific mortality among various PSCC SEER registries [26]. Here, we use the SEER database to report the up‐to‐date efficacy of site‐specific tumor resection on 5‐year overall survival (5y OS) among PSCC patients. We then report 5‐year survival outcomes in stage‐stratified patients with various numbers of regional lymph nodes biopsied and dissected; to our knowledge, we present the first SEER study to investigate these lymph node metrics in relation to overall survival.

## 2 Methods

### 2.1 Cohort Selection

Using the histological broad grouping of “squamous” with a primary site of origin of “penis”, we queried the SEER database to identify patients with PSCC. Variables assessed include patient demographic information, treatment modality, number of lymph nodes (LN) examined and/or dissected, disease stage/extent of disease and grade.

### 2.2 Retrospective Cohort Characterization

Patient demographics and tumor characteristics were recorded for the 5219 penile SCC patients at large spanning from 2000 to 2018. Demographics included age, sex, race, and marital status. Tumor characteristics included histologic type, stage, and grade. Age was subdivided into three categories surrounding the 50–70 age range, as patients in this range are at the highest risk for developing penile SCC.

Treatment characteristics included receipt of site‐specific surgery, radiation therapy, chemotherapy, and lymphadenectomy. Lymphadenectomy was further analyzed by the number of lymph nodes dissected based on SEER nodal grouping conventions, namely, no nodes dissected, one to three nodes dissected, or ≥ four nodes dissected; the SEER database readily reports this information according to the variable “RX Summ—Scope Reg LN Sur (2003+).” Prevalence of systemic therapy and radiation was stratified by tumor stage using SEER staging conventions, namely, localized, regional, distant, or unknown stage.

### 2.3 Statistical Analyses

Cohorts were characterized using descriptive statistics. Multivariate analysis involving demographic and treatment characteristics was carried out using Cox regression. Survival of various sub‐cohorts over 5‐year periods was compared via Kaplan–Meier survival analysis. A one‐sided *p*‐value < 0.05 on log‐rank tests was deemed statistically significant for all survival curves. Confidence intervals (CI) were set at 95%. Radiation and chemotherapy survival outcomes were stratified by tumor stage using SEER staging conventions. The number of lymph nodes biopsied and dissected was also stratified in this fashion.

Surgical status was categorized as excisional biopsy, local tumor excision, simple/partial removal, or no surgery. Radiation therapy and chemotherapy receipt were used as binary variables: either patients were confirmed to have received treatment or not. Timing of radiation or chemotherapy relative to surgery was classified as before, after, or before and after surgery. Numbers of nodes biopsied and dissected were both grouped by SEER nodal grouping conventions for lymphadenectomy. To create a binary analysis, we combined the no nodes biopsied or dissected and one to three nodes biopsied or examined into a single category. Marital status was categorized as married or unmarried. Clinical and pathologic grades were divided into grades I, II, III, and unknown.

## 3 Results

**FIGURE 1:**
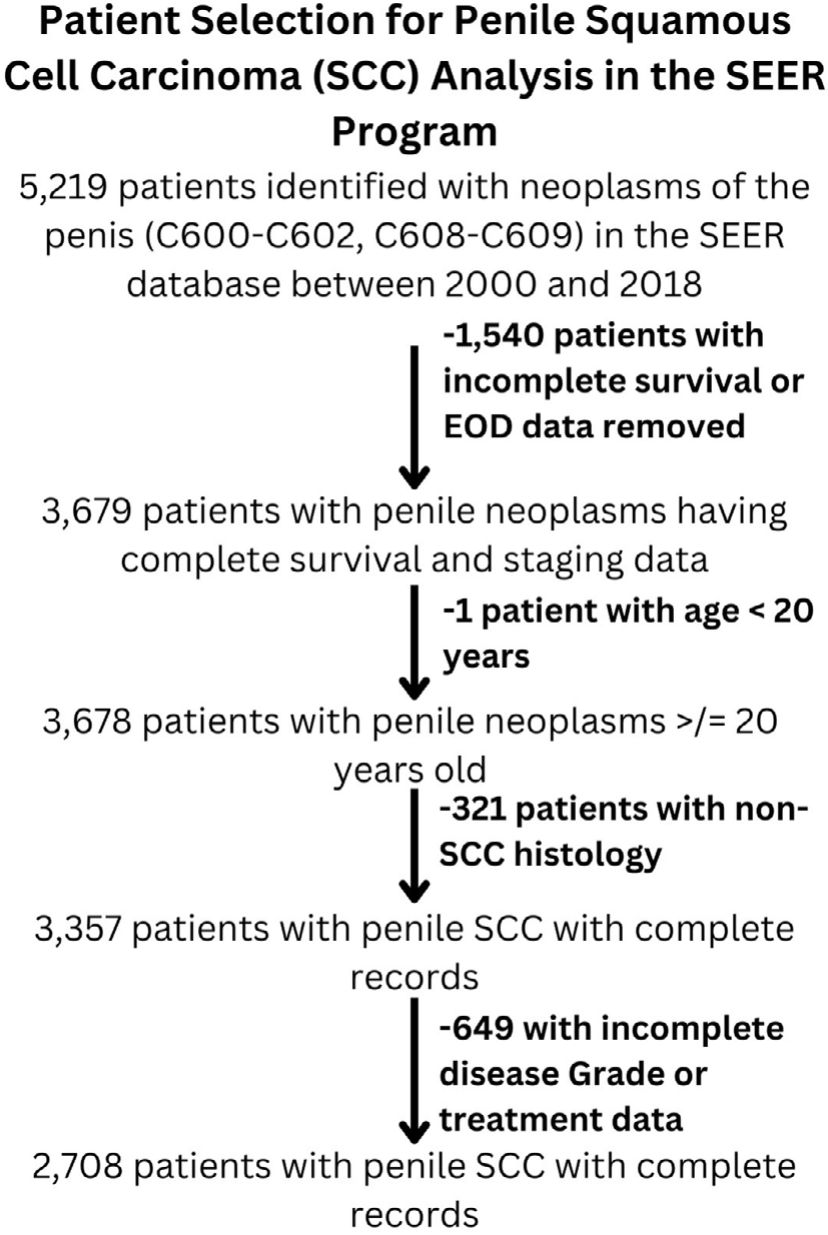
Algorithm utilized to parse patients identified with penile squamous cell carcinoma identified via the SEER database. Patients identified via the SEER database as having penile SCC were subsequently parsed to omit patients from analysis that were lacking complete variable data or that did not have SCC‐type histology. EOD, extent of disease (summary stage).

An initial cohort of 5219 patients diagnosed with neoplasms of the penis (C600‐C602 and C608‐609) between 2000 and 2018 was identified. Patients with incomplete survival, summary stagegrade, or treatment data were subsequently removed from this analysis, as were those < 20 years of age and those with non‐SCC histologies. Two thousand seven hundred eight patients remained after this parsing; a flowchart depicting this process can be found in Figure 1.

### 3.1 Cohort Characteristics

A breakdown by treatment and non‐treatment variables can be found in Table 1. All patients were male. A histogram depicting patient age can be found in Figure S1. Generally speaking, 57.8% of patients identified were ≥ 65 years at diagnosis, 54.9% were married at the time of diagnosis, 55.4% had local disease, and 82.9% were White. 50.8% had Grade II disease and only 21.6% had lymph nodes examined. The most common type of surgery was partial surgical removal of the identified cancer, followed by 17.5% of patients undergoing an excisional biopsy. 8.2% of patients underwent radiotherapy and 9.5% received chemotherapy. 7.4% received both chemotherapy and surgery and 7.0% received both surgery and radiotherapy.

**TABLE 1:** Cohort variables of patients with squamous cell carcinoma of the penis diagnosed between 2000 and 2018 (*n* = 2708).

Variable	Number (%)
Age (at diagnosis)	
< 65 years	1142 (42.2%)
≥ 65 years	1566 (57.8%)
Marital status	
Divorced	243 (9.0%)
Married	1487 (54.9%)
Separated	23 (0.8%)
Single	485 (17.9%)
Domestic partner	1 (0.0%)
Widowed	274 (10.1%)
Unknown	195 (7.2%)
Summary stage	
Local	1501 (55.4%)
Regional	1109 (41.0%)
Distant	98 (3.6%)
Race	
White	2244 (82.9%)
Black	323 (11.9%)
Asian or Pacific Islander	100 (3.7%)
American Indian/Alaska Native	29 (1.1%)
Unknown	12 (0.4%)
Histology	
SCC	2014 (74.4%)
Large cell SCC^a^	37 (1.4%)
Small cell SCC^a^	2 (0.1%)
Adenoid^a^	1 (0.0%)
Keratinizing^a^	637 (23.5%)
Micro‐invasive^a^	5 (0.2%)
Spindle cell^a^	11 (0.4%)
Grade	
Grade I (Well Differentiated)	701 (25.9%)
Grade II (Moderately Differentiated)	1375 (50.8%)
Grade III (Poorly Differentiated)	617 (22.8%)
Grade IV (Undifferentiated; Anaplastic)	15 (0.6%)
Lymph Nodes (LN) Examined	
Yes	586 (21.6%)
No	2008 (74.2%)
Aspiration Performed	41 (1.5%)
Unknown	73 (2.7%)
LN Positive	
Positive	329 (12.1%)
Negative	285 (10.5%)
Unknown	2094 (77.3%)
Regional LN Surgical Removal	
1–3	71 (2.6%)
≥ 4	448 (16.5%)
Sentinel LN biopsy	21 (0.8%)
None	1733 (64.0%)
Unknown	414 (15.3%)
Definitive Surgical Treatment	
Excisional Biopsy	475 (17.5%)
Electrocautery	13 (0.5%)
Laser Ablation/Excision	8 (0.3%)
Local Tumor Excision/Destruction	116 (4.3%)
Partial Surgical Removal	1454 (53.7%)
Radical Surgery	106 (3.9%)
Total Surgical Removal	379 (14.0%)
None^b^	157 (5.8%)
Radiotherapy (RT)	
External Beam	217 (8.0%)
Radioisotopes or Brachytherapy	6 (0.2%)
None/Unknown	2485 (91.8%)
Chemotherapy (CTX)	
Yes	258 (9.5%)
No/Unknown	2450 (90.5%)
Surgery and Systemic Therapy	
Adjuvant Systemic Therapy	171 (6.3%)
Neoadjuvant Systemic	18 (0.7%)
Neoadjuvant and Adjuvant Systemic	4 (0.1%)
Adjuvant and Neoadjuvant Surgery	8 (0.3%)
Unknown	715 (26.4%)
No Combination Given	1792 (66.2%)
Surgery and RT	
Adjuvant RT	182 (6.7%)
Neoadjuvant RT	5 (0.2%)
Surgery Before and After RT	1 (0.0%)
Intraoperative RT	2 (0.1%)
Unknown	3 (0.1%)
No Combination Given	2515 (92.9%)

*Note:* Percentages refer to parts of the entire 2708‐patient cohort.

^a^ Subtype of SCC.

^b^ Either non‐definitive surgical treatment (debulking surgery) or no surgery performed.

### 3.2 Multivariate Analysis

Cox regression analysis based on patient 5‐year overall survival (5y OS) was performed for the variables assessed in Table 1; these results can be found in Table 2. Patients with low disease summary stage (local > regional > distant), age < 65 years at diagnosis vs. age ≥ 65 years, lower disease Grade (I > II > III), and those that were married at the time of diagnosis with PSCC saw increased survival (*p* < 0.001, < 0.001, < 0.001, and 0.026, respectively). Those treated with CTX or RT saw decreased 5y OS (*p* < 0.001 and *p* = 0.002, respectively). It should be noted that for radiotherapy and chemotherapy, the variable was stratified in “yes” and “no/unknown,” as this is how SEER specifically breaks this variable down.

**TABLE 2:** Results from multivariate analysis of patient demographic and treatment variables for patients diagnosed with penile squamous cell carcinoma between 2000 and 2018.

Variable	Hazard ratio	*p*
Stage		< 0.001
Definitive surgery Y/N	1.006	0.954
Age		< 0.001
Race		0.136
Histology		0.615
Grade		< 0.001
Radiotherapy Y/N		0.002
Chemotherapy Y/N	1.502	< 0.001
Marital status at diagnosis		0.398

*Note:* Hazard ratios are provided for comparisons involving binary variables.

### 3.3 Summary Stage, Grade, and Histology Survival Analysis

Univariate survival analysis of summary stage demonstrated decreasing 5y OS conducive with increasing staging, with patients with local disease showing the highest survival (83% vs. 61% for those with regional disease and 15% for those with distant disease; *p* < 0.001). Similarly, patient 5y OS was inversely proportional to disease grade: Grade I = 86%, Grade II = 70%, Grade III = 59%, and Grade IV = 36% (*p* < 0.001). Stratification by disease histology and excluding histologies with less than 10 cases (parsing out one case of the adenoid SCC variant, five cases of the micro‐invasive variant, and two cases of the small cell variant) demonstrated patients with large cell and spindle cell variants of SCC had decreased 5y OS compared to both the keratinizing variant and patients coded as SCC (with no variant; 59% and 55% vs. 69% and 73%, respectively; *p* = 0.015).

### 3.4 Survival Analysis of Lymph Node (LN) Biopsy and Positivity

Excluding patients in which LN examination status was unknown (*n* = 73), patients that did not have LN examined saw increased 5y OS compared to those that had any number of LN biopsied and those that had final needle aspiration (FNA) of LN (76% vs. 66% and 37%, respectively; *p* < 0.001). This remained true for comparison of those that did not have LN examined to only those that had any LN biopsied (*p* < 0.001). Stratifying by stage, no difference was seen in 5y OS among these three groups for patients with local or distant disease (*p* = 0.736 and 0.539, respectively). Patients with regional disease that underwent FNA had decreased 5y OS compared to those that either had LN biopsied or those that did not (39% vs. 63% and 64% for those that had LN biopsied and those that did not, respectively; *p* = 0.004).

Of the patients with LN examined, patients with positive LN after examination saw decreased 5y OS compared to those with LN examined that subsequently were not positive for disease (82% vs. 52%; *p* < 0.001). This trend persisted among patients with local and regional disease (*p* = 0.070 and < 0.001, respectively). Patients that underwent FNA were more likely to have a positive result compared to those that underwent LN biopsy (76% vs. 56%; Likelihood Ratio = 10.4, exact, two‐sided *p* = 0.007). Among patients with regional disease this pattern remained: patients that underwent FNA saw increased rates of positive results compared to those that underwent biopsy (77% vs. 68%; exact, two‐sided *p* = 0.045). Accounting for stage, patients with regional disease who underwent LN biopsy saw increased 5y OS compared to those with positive FNA (53% vs. 29%; *p* < 0.001). No difference was seen among patients with local or regional disease (*p* = 0.650 and 0.287, respectively).

### 3.5 Demographic Analysis

Univariate survival analysis of patient demographic factors demonstrated patients who were identified as American Indian/Alaska Native and Asian or Pacific Islander (combined patient count of 129, or 7.8% of the entire cohort) both saw increased 5y OS compared to both White and Black patients (82% and 78% vs. 66% and 72%, respectively; *p* = 0.044). Additionally, Black patients saw decreased 5y OS compared to White patients (*p* = 0.024). Stratifying by stage, no difference in 5y OS was seen among races for patients with local or distant disease (*p* = 0.632 and 0.406, respectively). However, the same trend seen in the entire cohort was present among those with regional disease: American Indian/Alaska Native and Asian or Pacific Islander patients saw increased 5y OS (73% and 75%, respectively) compared to White and Black patients (62% and 48%, respectively; *p* = 0.015).

Patients < 65 years at diagnosis saw increased 5y OS compared to those ≥ 65 (74% vs. 70%; *p* = 0.010). The SEER database reports patient age in 5‐year intervals (e.g., 25–29 years, 30–34 years, etc.) with the oldest category including all patients 85 years and older. Patients 85 years or older at diagnosis saw the lowest 5y OS (61%) which was lower than the 5y OS for all other age intervals (*p* = 0.002).

Survival analysis based on patient marital status demonstrated patients listed as separated at the time of diagnosis had increased 5y OS compared to those that were married or single (85% vs. 74% and 71%, respectively). Those that were widowed or divorced saw the lowest 5y OS of the entire group (64% for both). Log‐rank analysis demonstrated these three groups (separated vs. married and single vs. widowed and divorced) had significantly different 5y OS (*p* = 0.016). No difference in 5y OS was seen between single and married patients (*p* = 0.178).

### 3.6 Treatment of Local or Regional, Low‐Grade PSCC

Among patients with local disease and either Grade I or II disease, those that underwent local excision (electrocautery, laser excision/ablation, etc.) saw increased 5y OS compared to those that underwent partial or total penectomy (90% vs. 85% and 69%, respectively; Log Rank *p* < 0.001). Specifically, minimally invasive surgery demonstrated increased survival when compared to partial penectomy (*p* = 0.019). All patients treated with external beam radiotherapy (EBRT) alone died within 4 years of diagnosis.

For patients with regional disease, those that underwent local excision saw improved 5y OS compared to those that were treated with a total penectomy (90% vs. 69%, respectively; *p* = 0.030). Local excision was noninferior to partial penectomy (82% vs. 70%, respectively; *p* = 0.167). No patients were treated with EBRT alone; the addition of chemotherapy to EBRT was noninferior to total penectomy (*n* = 9; 39% vs. 62%, respectively; *p* = 0.160).

### 3.7 Treatment of Local or Regional, High‐Grade Disease

Among patients with local disease and either Grade III or Grade IV disease, those that underwent local excision saw increased 5y OS compared to both partial and total penectomy (92% vs. 70% and 72%, respectively; *p* = 0.008). No patients were treated with EBRT alone and all patients treated with EBRT in addition to chemotherapy died within 1 year of diagnosis (*n* = 10).

For patients with regional disease, no difference in survival was seen among those treated with local excision compared to partial or total penectomy (52% vs. 54% and 61%, respectively; *p* = 0.855). Those treated with EBRT alone saw decreased 5y OS (*n* = 6) compared to local excision (17% vs. 52%, respectively; *p* = 0.089). The addition of chemotherapy to EBRT was noninferior to surgical treatment (62% vs. 52%, respectively; *p* = 0.536).

### 3.8 LN Dissection

Comparing patients that underwent LN dissection to those that did not, no difference was seen among those that had any number of LN dissected compared to those that did not for patients with local or distant disease (*p* = 0.931 and 0.364, respectively). For patients with regional disease, those with four or more LN dissected saw increased 5y OS compared to those that did not (69% vs. 61%, respectively; *p* = 0.002). Those that had one to three LN dissected saw the lowest 5y OS (41%; *p* = 0.009 compared to those that did not have LN dissection).

### 3.9 Comparison of Invasive Surgical Methods

Patients with local disease that underwent partial penectomy alone saw 5y OS noninferior to those that underwent radical penectomy (82% vs. 88%, respectively; *p* = 0.759). Those that underwent partial penectomy had improved 5y OS compared to those that were treated with a total penectomy (82% vs. 70%, respectively; *p* = 0.003). No difference was seen among invasive surgical methods in patients with regional disease (*p* = 0.196).

## 4 Discussion

Penile cancer is a devastating cancer in the developing world, with penile SCC composing the vast majority of these cancers. While it has been reported that males aged 50–70 years are at the highest risk of acquiring penile SCC [1], our study showed a slightly higher prevalence in men older than 70, though the number of cases increased significantly past 50 years of age (Table S1 and Figure S1). This may be more of a representation of the United States compared to worldwide populations.

Surgical resection, including excisional biopsy, occurred in 2551 of the 2708 patients identified (94.2% of the entire cohort), which concurs with literature recommending surgical management primarily in most patients. When stratified by treatment with definitive surgery during Cox regression for 5y OS though, no benefit was seen with surgical treatment (*p* = 0.95). This is in comparison to treatment both with chemotherapy and radiotherapy, which as standalone variables were associated with decreased 5y OS (*p* < 0.001 and = 0.002, respectively). Interpretation of this is somewhat limited, however, as this does not take into account any other factors such as disease grade or stage, which influence the type of surgery performed. Grade itself was inversely associated with increasing 5y OS on multivariate analysis. Similarly, multivariate analysis of 5y OS based on disease histology was inconclusive for the entire cohort but interpretation is limited given the significant number of cases with histologies of PSCC with fewer than 10 cases.

Given the limited interpretation possible from the initial multivariate analysis conducted, a subanalysis was conducted on a univariate basis. This demonstrated decreasing survival with increasing disease stage and grade, in keeping with the literature [1] and common sense. Univariate analysis did demonstrate decreased survival among those patients with keratinizing and spindle cell SCC variants compared to others, though again this analysis required the omission of multiple histologies with fewer than 10 cases in the entire cohort. Future work can potentially explore the association between histology and survival among patients with PSCC further.

Disease grade plays a role in the treatment appropriate for PSCC, with higher grade disease warranting more aggressive therapy, generally [1, 14]. Subanalysis regarding grade among those patients with local or regional disease, was thus explored. Among those patients with local, low‐grade (Grade I or II) disease, those that underwent local excision had increased 5y OS compared to those that underwent partial or total penectomy. This may be a reflection of both patient selection, though it does raise the question of potential increased utilization of less invasive surgical methods among patients with PSCC. The same was true among patients with regional, low‐grade disease. The addition of radiotherapy and in the case of regional disease chemotherapy, was not associated with increased survival, though this analysis was somewhat limited by the number of available cases. Our analysis of overall survival with disease stage was roughly congruent with previous literature [27].

Similarly, subanalysis of patients with local or regional but high‐grade disease demonstrated that those with high‐grade, local disease had increased 5y OS with treatment with local excision compared to partial or total penectomy. No difference was seen however, among those with regional, high‐grade disease. Radiotherapy was also not associated with increased 5y OS among these patients. Previous literature has found that radiotherapy is inferior in terms of survival benefit for local disease not stratified by grade, though it should be noted that this was specifically for cause‐specific mortality [28].

Lymph node involvement is an important component of prognostication among patients with PSCC [1, 6]. This specific variable was omitted from multivariate analysis as it is tied to disease stage, though subanalysis was conducted regarding lymph node biopsy and positivity. Patients that did not have either LN biopsied or undergo fine needle aspiration had increased 5y OS on this analysis, though the authors interpret this as likely related to initial disease stage, with those with very localized disease likely not necessitating lymph node examination and also having increased 5y OS as a product of the more localized disease. We did test this with further analysis, and found no difference in 5y OS among those patients with LN examined or that underwent fine needle aspiration among patients with local or distant disease, though patients with regional disease that underwent fine needle aspiration did have decreased 5y OS (*p* = 0.004), which may itself be reflective of an area for improvement in more aggressive lymph node biopsy among these patients.

Interestingly, when analysis of 5y OS with LN positivity was conducted, in general fine needle aspiration was associated with increased rates of positivity, yet patients with positive LN biopsied had increased 5y OS. This further supports more aggressive lymph node biopsy lymphadenectomy, as suggested in the literature as well [12, 13]. Further subanalysis demonstrated that patients with ≥ 4 LN dissected had increased 5y OS compared to those with fewer, further supporting this inference.

Demographic multivariate analysis was not significant for race and marital status (*p* = 0.14 and 0.40, respectively). Stratifying by race on univariate analysis did demonstrate increased 5y OS among patients that were American Indian/Alaska Native or Pacific Islander, though these cohorts were much smaller than the White and Black groups. Our study found that 54.9% of patients that were married at the time of diagnosis saw no difference in 5y OS compared to those single at the time of diagnosis (*p* = 0.18). The lack of statistical difference in survival does run counter to previous literature [29, 30], though it is possible the strict parsing of variables and removal of incomplete entries modified this trend if it does exist in the data assessed. Additionally, and as noted later in the limitations paragraph, not having information on HPV status does significantly limit the interpretation of the marital status as HPV is a known precursor to SCC of the penis [31].

Databases such as SEER possess inherent limitations, namely, difficulty decoding variable groups, blank data entries, lack of clear staging and grading data, and a lack of risk‐based variables, such as HPV status. It should be noted especially that exposure to tobacco and HPV are known contributing factors to penile SCC. Expanding penile SCC histological subtypes in SEER would allow for comparisons between in situ disease, microinvasive disease, and disease with stromal invasion—a level of detail that is currently unavailable for all examined histologies, and, thus, not investigated in the present analysis. We also recognize the 5‐year time frame of our Kaplan–Meier survival curves limits the long‐term applications of our study. Finally, the decision to limit analysis to patients between 2000 and 2018 was two‐fold: the newest AJCC staging guidelines, (8th edition), published partway through this project's creation, and including patients using this new staging schema was not possible as few patients had been staged using this new system. Second, conversion from old to new staging is now somewhat difficult and could perhaps be misleading, as not only do the new AJCC staging guidelines affect T1 cancer, but the distinction between T2 and T3. This study was also limited in that a significant portion of the patients included lacked staging (21.3%) and grading (22.0%) information initially, and thus were parsed from the analysis; this is secondary to information reported in the SEER database and may be related to how patient information is reported to the SEER database. National efforts to improve the adequacy of reported cancer data would be a possible solution. It should also be noted that the choice to stratify lymph node removal at four was based on preexisting SEER variables; the choice to conduct our analysis using this cutoff may give the impression that lymph node removal does not affect survival, though it is possible at higher cutoffs lymphadenectomy may prove beneficial. Previous work has also explored regional differences in survival with PSCC, an area not explored in this project [26]. Finally, our analysis was limited in that multivariate analysis was not conducted; future analysis of penile SCC would benefit from this type of analysis, as comprehensive control for various factors such as stage, grade, and comorbidities is not possible with univariate analysis.

In summary, we conducted the first SEER study on overall survival outcomes for penile SCC patients based on quantities of lymph nodes biopsied and dissected across disease Stage; we demonstrate that the majority of the survival benefit of both lymph node biopsy and lymph node dissection of at least four lymph nodes occurs in the setting of regional disease. This finding has clinical relevance not only for surgeons, but also for pathologists as well, particularly at large academic institutions with higher case volumes. Our study reaffirms the value of local tumor excision and the superiority of neoadjuvant chemotherapy over adjuvant chemotherapy. Among patients with low‐grade disease, less invasive surgical methods were associated with increased survival. Among patients with high‐grade disease, less invasive surgical methods were associated with noninferior or improved survival depending on disease stage. Future investigation of both less invasive surgical methods and the increasing role of lymphadenectomy among patients with SCC of the penis is warranted.

## Author Contributions

Conceptualization: George M. Edwards, Andrew W. Ju, Arjun Bhatt. Methodology: Michael C. Larkins. Software: Michael C. Larkins. Data curation: Arjun Bhatt. Investigation: George M. Edwards, Lucas W. Ashley, Austin Holmes, Andrew W. Ju, Arjun Bhatt, Michael C. Larkins. Validation: Michael C. Larkins. Formal analysis: George M. Edwards, Lucas W. Ashley, Austin Holmes, Arjun Bhatt, Michael C. Larkins. Supervision: Arjun Bhatt, Michael C. Larkins. Funding acquisition: N/A. Visualization: Arjun Bhatt, Michael C. Larkins. Project administration: Austin Holmes, Arjun Bhatt, Michael C. Larkins. Resources: Michael C. Larkins. Writing – original draft: George M. Edwards, Lucas W. Ashley, Andrew W. Ju, Arjun Bhatt, Michael C. Larkins. Writing – review and editing: Michael C. Larkins. All authors listed above contributed substantially to the conception or design of this work, the drafting of the work and subsequent revisions, approved the final version to be published, and agreed to be accountable for all aspects of the work regarding accuracy and integrity.

## Disclosure

The authors declare that the research was conducted in the absence of any commercial or financial relationships that could be construed as a potential conflict of interest. Approval by an Institutional Review Board (IRB) is not applicable, as the data analyzed in this analysis are publicly available through the NCI SEER program. Similarly, registration for clinical trials and informed consent are not applicable to this project. Finally, this project does not involve the use or study of animals. The views and opinions expressed in this article/presentation are those of the author(s) and do not necessarily reflect official policy or position of the United States Air Force, Defense Health Agency, Department of Defense, or U.S. Government.

## Conflicts of Interest

The authors declare no conflicts of interest.

## Supporting information


**Figure S1:** Histogram depicting breakdown by age bracket of cases of squamous cell carcinoma of the penis, identified via the SEER database from 2000 to 2018. Age brackets are standard from the SEER database.

## Data Availability

The data that support the findings of this study are openly available in Surveillance, Epidemiology, and End Results (SEER) Program at https://seer.cancer.gov/.
