# Supplementary figures and images for: Demographics, Clinical Characteristics, and a Stage‐Based Analysis of Treatments and Outcomes for Squamous Cell Carcinoma of the Penis
Source: Cancer Rep (Hoboken). 2025 Dec 3;8(12):e70383. doi: 10.1002/cnr2.70383 (PMC12674586; doi:10.1002/cnr2.70383)

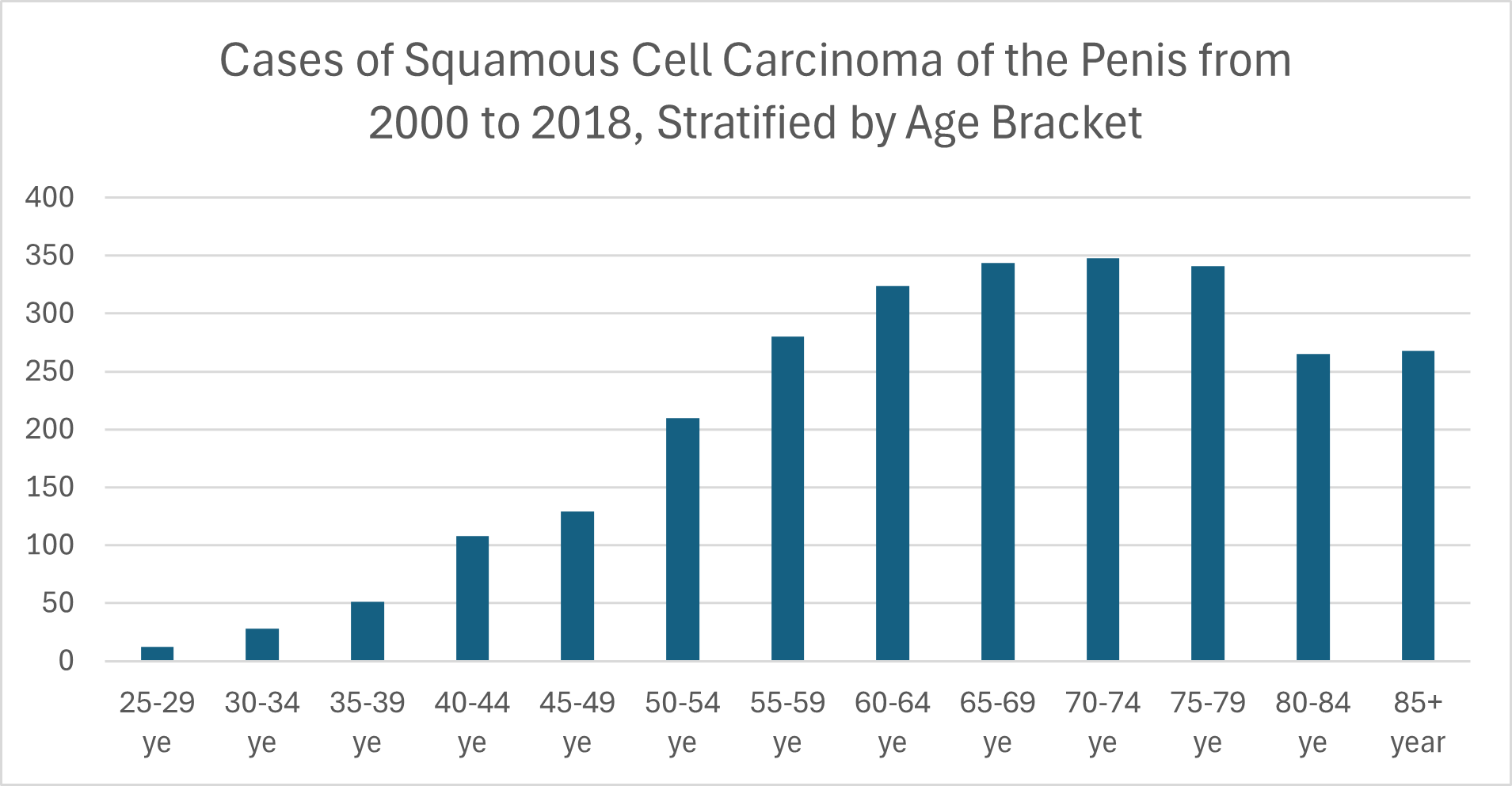

Supplement: Supplementary file 1 — Figure S1: Histogram depicting breakdown by age bracket of cases of squamous cell carcinoma of the penis, identified via the SEER database from 2000 to 2018. Age brackets are standard from the SEER database. [file CNR2-8-e70383-s001.png]
